# Dehydroascorbic acid sensitizes cancer cells to system x_c_^-^ inhibition-induced ferroptosis by promoting lipid droplet peroxidation

**DOI:** 10.1038/s41419-023-06153-9

**Published:** 2023-09-27

**Authors:** Luciano Ferrada, María José Barahona, Matías Vera, Brent R. Stockwell, Francisco Nualart

**Affiliations:** 1 Center for Advanced Microscopy CMA BIO BIO, Faculty of Biological Sciences, University of Concepcion, Concepcion, Chile; 2 Laboratory of Neurobiology and Stem Cells, NeuroCellT, Department of Cellular Biology, Faculty of Biological Sciences, University of Concepcion, Concepcion, Chile; 3 Department of Chemistry, Columbia University, New York, NY 10027 USA; 4 Department of Biological Sciences, Columbia University, New York, NY 10027 USA

**Keywords:** Cell death, Cancer therapeutic resistance, CNS cancer, Prostate cancer

## Abstract

Since the discovery of ferroptosis, it has been postulated that this type of cell death could be utilized in treatments for cancer. Unfortunately, several highly aggressive tumor models are resistant to the pharmacological induction of ferroptosis. However, with the use of combined therapies, it is possible to recover sensitivity to ferroptosis in certain cellular models. Here, we discovered that co-treatment with the metabolically stable ferroptosis inducer imidazole ketone erastin (IKE) and the oxidized form of vitamin C, dehydroascorbic acid (DHAA), is a powerful therapy that induces ferroptosis in tumor cells previously resistant to IKE-induced ferroptosis. We determined that DHAA and IKE + DHAA delocalize and deplete GPX4 in tumor cells, specifically inducing lipid droplet peroxidation, which leads to ferroptosis. Moreover, in vivo, IKE + DHAA has high efficacy with regard to the eradication of highly aggressive tumors such as glioblastomas. Thus, the use of IKE + DHAA could be an effective and safe therapy for the eradication of difficult-to-treat cancers.

## Introduction

Vitamin C is found in 2 redox states, reduced as ascorbic acid (AA) and oxidized as dehydroascorbic acid (DHAA) [1, 2]. It has been postulated that pharmacological doses of AA may have antitumor activity due to the extracellular production of H_2_O_2_, triggering conventional necrosis in cancer cells [3, 4]. More recently, it has been determined that the H_2_O_2_-iron-glutathione (GSH) axis plays a key role in the mechanism of induced death mediated by pharmacological doses of AA [5, 6], ruling out DHAA as a death inducer, since it does not generate H_2_O_2_ in solution [5, 7]. These antecedents led to the proposal that there could be a crossroad between ferroptosis and cell death induced by pharmacological doses of AA.

Ferroptosis is a novel type of nonapoptotic cell death dependent on iron and glutathione peroxidase 4 (GPX4) enzyme activity, which is triggered by an overwhelming overload of lipid ROS and can be inhibited by radical-trapping antioxidants such as ferrostatin-1 (Fer) or the vitamin E analog Trolox, among others [8, 9]. The most studied classical pathway of ferroptosis induction can be induced by the small molecule erastin or its metabolically stable analog imidazole ketone erastin (IKE), which inhibits system x_c_^-^ [10]. System x_c_^-^ is a cystine antiporter formed by the subunits SLC7A11, which acts as a transporter, and SLC3A2, which acts as an anchor to the plasma membrane [9, 11]. Thus, the inhibition of system x_c_^-^ by erastin/IKE triggers cysteine starvation in the cell and the concomitant depletion of GSH. Furthermore, GSH is necessary to maintain GPX4 activity; therefore, treatment with erastin/IKE triggers ferroptosis due to GPX4 inactivation by GSH depletion [9].

Despite the possible link between ferroptosis and AA, when tumor cells are treated with pharmacological doses of AA, cell death is induced and cannot be rescued by classic ferroptosis inhibitors such as Fer [12, 13], ruling out a possible direct connection between ferroptosis and cell death induced by pharmacological doses of AA. On the other hand, treatment with DHAA can induce tumor death due to GSH consumption and ROS production [14], similar to the effects induced by IKE [9, 10]. This evidence raises an intriguing hypothesis: co-treatment with DHAA and erastin or IKE favors the induction of ferroptosis in cancer cells because both molecules are closely related in tumor cell death-induction mechanisms.

Here, we tested this hypothesis through various pharmacological approaches coupled to high-content kinetic cell death assays using super-resolution real-time live-cell microscopy and an in vivo xenograft model, revealing a potent and unrecognized coupling between IKE and DHAA that induces ferroptosis in tumor models otherwise resistant to IKE-induced ferroptosis. We found that IKE + DHAA specifically induce lipid droplet peroxidation, a key event sufficient to trigger ferroptosis in IKE-resistant cancer cell. In this context, DHAA synergizes with IKE because it favors GSH consumption and delocalization of GPX4 from lipid droplets. The results support the conclusion that co-treatment with IKE + DHAA could be useful in the eradication of difficult-to-treat cancers.

## Material and methods

The reagents are described in Supplementary Table 1.

### Mice

Mice were treated in compliance with the U.S. National Institutes of Health guidelines for animal care and use, c57bl/6j mice strain were used in this study. Xenograft experiments were performed in 8 - to 12-week-old male mice. Animals were maintained at 23 °C, with an 12 h light/dark cycle (07.00 a.m on –07.00 p.m) and a 40% ambient humidity. 3–5 mice were housed per cage and for each experiment they were randomly chosen. Mice were fed *ad libitum* a standard diet for rodents (Lab Diet 5001 rodent diet). It is important to mention that all the experiments were reviewed and approved by the bioethics committee of the Universidad de Concepción and by the national research and development agency (ANID, protocols for project N°11200335).

### Stereotaxic surgery

Mice were anesthetized with isoflurane and placed in the stereotaxic frame (RWD, # 68037). Then, with the help of a scalpel, an antero-posterior incision was made, and the skull was exposed. Next, a cannula guide (20 Gauge Stainless-Steel, P1 Technologies Inc) was implanted in the right lateral ventricle following the bregma coordinates AP: −0.11 mm, ML: −1.0 mm and DV: −2.2 mm. After implantation, the cannula guide was fixed to the skull with cyanoacrylate gel. (P1 Technologies Inc). The procedures were performed by the same surgeon and previously published asepsis and analgesia protocols were followed [15]. Mice were allowed to recover for 5 days prior to xenograft generation.

### Xenograft generation

After 5 days of post-stereotaxic recovery [15], mice were randomly chosen and briefly anesthetized with isoflurane. Next, using an intracerebroventricular (icv) single internal cannula (P1 Technologies Inc) and a Hamilton syringe, 4 µl containing 500,000 U87 cells were administered into the right lateral ventricle. The tumour was allowed to grow for 7 days post-injection.

### Convection-enhanced delivery

7 days after the xenograft generation, the convection-enhanced delivery was carried out. The drugs were administered daily (09:00 a.m.) for a period of 7 days, giving rise to 5 experimental groups: Group 1 (control), was injected icv with 4 µl of PBS. Group 2 was injected icv with 4 µl of DHAA (20μg).

Group 3 was injected icv with 4 µl of IKE (650 ng). Group 4 was treated icv with 4 µl of IKE (650 ng) + DHAA (20 µg). Finally, group 5 was treated icv with 4 µl of IKE (650 ng) + DHAA (20 µg) + (Liproxtatin) Lpx (20 ng). Mice were sacrificed the next day after receiving the last icv dose.

### Hematoxylin and eosin stain

Mice were anesthetized with isoflurane and were transcardially cannulated. Subsequently, it was washed with PBS and fixed by vascular perfusion with 4% paraformaldehyde (PFA). Subsequently, the brains were collected and post-fixed for 24 h before being embedded in paraffin. Frontal sections of 7 µm were obtained using a microtome (Leica, RM2125 RTS) and the slice were mounted on slides treated with poly-lysine (0.1 mg/ml) (Sigma-aldrich). Subsequently, each brain section was deparaffinized and hematoxylin and eosin stained (Sigma-aldrich). A Nikon Eclipse Ni microscope (Nikon) with brightfield optics at 4x magnification was used to analyze the sections. Tumor area (µm^2^) was calculated using ImageJ software. For the study of tumor invasion, photos were taken from the anterior region of the lateral ventricles to their posterior region. Next, the number of mm covered by the tumor in the anterior-posterior axis was quantified with the help of a mouse stereotaxic atlas. Invasion at third ventricle (3V) was calculated as the percentage of the number of animals that presented invasion. The ventricle area (µm^2^) was calculated using ImageJ software. The photographs of the intracranial brain tumor were obtained using a stereo microscope (Nikon SMZ18).

### Immunohistochemistry

Brains were fixed as previously described. Next, 3 washes of 10 min each were performed using the Tris-Phosphate buffer. Subsequently, the slices were incubated with the primary antibody: Anti-Vimentin (1:400), Anti-Nestin (1:200), Anti-GFAP (1:200) and Anti-3F3-FMA (1:200), anti-MDA (1:100) throughout the overnight. Then, samples were washed 3 times for 10 min with Tris-phosphate buffer and incubated with the following secondary antibodies: Anti-Mouse Cy5, Anti-Chicken Cy3 and Anti-Rabbit cy2 (The Jackson Laboratory) for 2 h. Hoechst 33342 (1:1000) was used for nuclear staining. Finally, the sections were mounted using fluorescence mounting medium (Dako).

### Primary and cell line culture

Neural stem cell cultures have been previously reported by us [16, 17]. U87 and HeLa cells were maintained in DMEM high-glucose (HyClone #SH30081.02). HSVT-C3 and BTIC-13 cells were maintained in medium DMEM/F-12 (GIBCO #11320033). Primary neural stem cells were maintained in proliferation medium (Stem Cell Technologies) supplemented with epidermal growth factor (EGF; 20 ng/mL), fibroblast growth factor (FGF; 10 ng/mL) and heparin (10 ng/mL) (Stem Cell Technologies). LNCaP, C4-2B, 22Rv1, DU-145 and ZR-75-30 cells were maintained in medium RPMI 1640 (HyClone #SH30255.02). Human dermal fibroblasts (HDF) were cultured in MEM (HyClone #SH30024.02). All cells were supplemented with 10% FBS, except HSVT-C3 and HDF cells which were supplemented with 5% FBS (Mediatech, #MD.35-010-CV), GlutaMAX (GIBCO # 35050061) and 100 U/mL penicillin-streptomycin (Corning # 30-002-CI). To ensure mycoplasma-free cultures, the cells were treated with BM cyclin (Roche, #10799050001). Cells were maintained at 37 °C, 5% v/v CO2 in a humidified incubator. Importantly, all cells were used with passage no greater than 30 or 35, because aged cultures showed dissimilar responses and increased sensitivity to ferroptosis inducers such as IKE and RSL-3.

### Compound treatment in cell culture

In all experiments, a single dose of the ferroptosis-inducing compound was used at the concentration indicated in the figure. In all cases, except where otherwise indicated in the figure, a concentration of 1 mM DHAA plus 5 µM IKE for U87 cells or 2 µM IKE for 22Rv1 cells was used. The treatment with fresh AA and DHAA was performed in a single dose. Diacylglycerol O-acyltransferase 1 (DGAT1) inhibition with DGAT1i was performed for 48 h prior to treatment with IKE + DHAA. Cells were pre-incubated with buthionine sulfoximine (BSO) for 24 h prior to DHAA treatment.

### IncuCyte cell by cell death kinetic assays

For death assays, cells were seeded in 96-well plates with 2500 to 5000 cells per well overnight. The next day, the incubation with the compounds (Table S1) and sytox green (30 nM) was performed automatically using the OT-2 robotic platform (Opentrons). Serial dilution curves were made with a dilution factor of 2 automatically with the OT-2. Once the plates were in their respective experimental conditions, they were placed in the IncuCyte S3 real-time monitoring system. Subsequently, the IncuCyte was configured to acquire 2 images per well with the 10x objective, in phase contrast and the FITC channel, every 1 or 2 h for up to 48 h. Kinetic death analyzes were performed automatically every 1 or 2 h with the “cell by cell” processing and segmentation module (Sartorius # 9600-0031), where the number of dead cells (Sytox^+^) was constantly divided by the total number of cells in each image. Thus, the percentage of cell death was determined continuously over time.

### Real-time 3D single spheroid assays

U87 (1500 cells per well) and 22Rv1 (1000 cells per well) cells were seeded in 96-well ultra-low attachment plates (Costar #7007) and centrifuged at 2000 RPM for 10 min. Then, the cell aggregates were corroborated by microscopic observation and the formation of spheroids was allowed for 4 days. At day 5, the cells were treated with the compounds and the medium was supplemented with Sytox green to determine the rate of death. The size and death of the spheroids was automatically quantified every 2 or 3 h with the IncuCyte system coupled to the Spheroid Analysis Software Module (Sartorius #9600-0019). Cell death was expressed relative to Sytox fluorescence in green calibrated Unit (GCU) by μm^2^.

### Measurement of general ROS, lipid ROS and mitochondrial ROS

300,000 cells seeded in 6-well plates were treated with DMSO, DHAA, IKE or IKE + DHAA in the presence/absence of Fer for 10 or 12 h. Subsequently, the cells were trypsinized and resuspended in medium where they were incubated with 500 nM of CellRox deepRed (General ROS sensor), 5 μM of Image-iT Lipid Peroxidation Sensor (Lipid ROS sensor) or 5 μM of MitoSOX (Mitochondrial ROS Sensor) for 30 min at 37 °C. Then, the cells were washed 3 times with PBS by centrifugation and analyzed by flow cytometry (BD FACSAria III). The flow cytometry data were processed with FlowJo software (Tree Star).

### GSH assay

400,000 cells were seeded in 6-well plates. 7 h after treatment (DMSO, DHAA, IKE or IKE + DHAA) the medium was removed, a PBS wash was performed quickly and the cells were immediately placed in sulfosalicylic Acid. Then, the manufacturer’s instructions for quantifying GSH levels according to ab239727 Kit were followed.

### Quantitative PCR analysis

Total RNA from 400,000 cells per well was isolated using Trizol reagent according to the manufacturer’s instructions. RNA reverse transcription was performed with the high-capacity cDNA reverse transcription kit according to the manufacturer’s instructions. The sets of primers used are listed in table S1. The real-time PCR mix was prepared in hard-shell^®^ 96-Well PCR Plates, low profile (BioRad #HSP9601). The master mix (Brilliant II SYBR® Agilent Technologies) and the cDNA sample were dispensed automatically with the OT-2 (Opentrons). PCR (95 °C for 10 min; 95 °C for 30 s; 60 °C for 20 s; 72 °C for 20 s; 40 cycles) was carried out in Mastercycler Realplex^2^ (Eppendorf). The relative expression of mRNA to cyclophilin was calculated using the 2^−ΔΔCt^ method.

### Immunoblotting

800,000 cells were treated with DMSO, DHAA, IKE or IKE + DHAA for 5 or 7 h. Subsequently, cells were lysed for 30 min at 4 °C and agitation in RIPA buffer (Thermo Fisher Scientific #89901) supplemented with Protease/Phosphatase Inhibitor Cocktail (cell signaling #5872). The homogenate was centrifuged at 12,000 rpm for 15 min at 4 °C and the supernatant was collected for analysis and protein concentration was determined by the Bradford method. 20 μg of proteins were loaded and separated on TGX FastCast Acrylamide 10% gels (BioRad #1610173). Then, the proteins were transferred to PVDF membranes (0.45 μm pore; Immobilon-P #IPVH00010, Merck Millipore) and probed with anti-GPX4 in 1:100 dilution overnight at 4 °C. The next day, the membrane was incubated with HRP secondary antibody (1:5000) and the reaction was developed using the Western Lighting® Plus-ECL enhanced chemiluminescence substrate (PerkinElmer #NEL103001EA). Anti-actin conjugated to HRP was used 1:20,000 and incubated for 2 h at room temperature.

### Lightning super resolution live cell microscopy

2500 to 5000 cells were seeded per well in cellview cell culture slide, glass bottom, advanced TC (Greiner Bio-One #543979) overnight. Then, the cells were treated with the experimental conditions for 5 or 7 h. After treatment, cells were incubated with quadruple staining to stain the plasma membrane (Cellmask stain 0.3X), the nucleus (Hoechst 33342, 0.1 μg/mL), lipid peroxidation (Image-iT™ lipid peroxidation kit -based on C-11 Bodipy, 3 μM) or lipid droplets (LipidSpot 610 1X) for 30 min at 37 °C. After the incubation time with the probes, the cells were washed with PBS twice, incubated in complete medium and monitored in the Leica SP8 microscope equipped with a Lightning super resolution, CO_2_ and temperature control module, 3 ultrasensitive hybrid detectors (HyD) to avoid phototoxicity and photobleaching. The images were acquired in *x,y,z* at a size of 1024×1024 and are represented as projection of maximum intensity generated in the LASX software. For the analysis of U87 cells, the HC PL APO 40x/1,30 OIL CS2 oil immersion objective was used. For the analysis of 22Rv1 cells, the HC PL APO 63x/1,40 OIL CS2 oil immersion objective was used.

### Immunocytochemistry

Cells were seeded on coverslips. After treatment, the cells were fixed with 4% paraformaldehyde for 30 min at room temperature, washed with Tris-phosphate buffer and incubated overnight at room temperature with the following antibodies Anti-GPX4 (1:100), ani-3F3-FMA (1:100) and anti-Drp1 (1:100) or Anti-vimentin (1:100) overnight. For mitochondrial staining, mitotracker red CMXRos (100 nM 20 min, before fixation) was used. The next day, the cells were washed 3 times with Tris-phosphate buffer and incubated with the respective secondary antibodies (The Jackson Laboratory) for 2 h. Hoechst 33342 (1:1000) was used for nuclear staining. The images were acquired in the Leica SP8 microscope in confocal mode with HC PL APO 63x/1.40 OIL CS2 oil immersion objective and post-processed with the Lightning super resolution module. The images were exported in .lif format and processed in Imaris v 9.1 software (Bitplane Inc) for 3D reconstruction and mitochondrial volume analysis.

### esiRNA knockdown

U87 and 22Rv1 cells were grown to 70% confluence confirmed by IncuCyte analysis. The transfection of the pre-designed MISSION esiRNA (Merck) was performed with RNAiMAX (Invitrogen) according to the manufacturer’s instructions, with a final concentration of 30 pmol of esiRNA per well in 6-well plates. As a control, an esiRNA against RLUC (#EHURLUC) was used. After 48 h of incubation, the knockdown of *Dgat1* was validated by quantitative PCR. Subsequently, 48 h after validation of the knockdown, the cells were seeded in 96-well plates at a density of 1500 cells per well overnight (since at higher confluence it was observed that lipofectamine induces resistance to ferroptosis, similar to other published data [18]), and cell death analyzes were performed by IncuCyte with DMSO, IKE and IKE + DHAA treatments.

### Statistical analysis

Statistical tests were done using GraphPad Prism software. The statistical test used, and *p* value interval considered significant were indicated within each figure. When comparisons were between two groups, standard Student’s *t* test was used. For comparison of more than two groups, standard one-way ANOVA was used. In all cases normal distributions were assumed, and *p* values less than 0.05 were considered statistically significant.

## Results

### Co-treatment with IKE and the oxidized form of vitamin C, DHAA, sensitizes tumor cells to ferroptosis

**Fig. 1:**
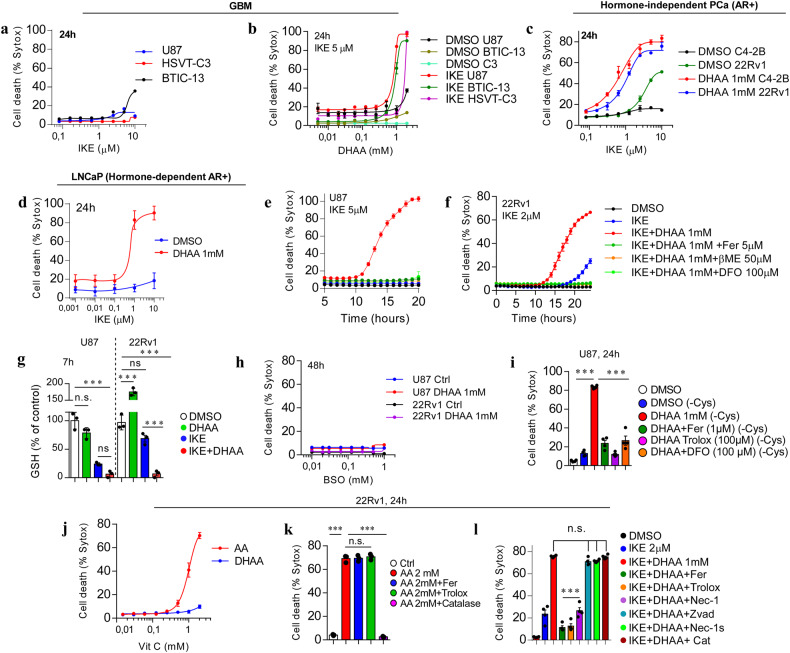
IKE + DHAA treatment induces ferroptosis in GBM and PCa models. **a** IncuCyte dose-response curve analysis showing that GBM cells exhibit resistance to IKE-induced ferroptosis. **b** IncuCyte dose-response curve analysis showing that GBM cells can be sensitized to death by IKE + DHAA co-treatment. **c**, **d** Dose-response curve of the effect of IKE plus DHAA co-treatment on cell death PCa models determined by IncuCyte. **e**, **f** Incucyte time-plot showing that IKE + DHAA induces ferroptosis in GBM and PCa. **g** Quantification of GSH levels. **h** BSO + DHAA fails to induce ferroptosis in GBM and PCa. **i** Cyst(e)ine depletion and DHAA co-treatment recapitulates the effects of IKE + DHAA to induce ferroptosis. **j** IncuCyte analysis showing that pharmacological doses of AA, but not DHAA, induce cell death in 22Rv1 cells. **k** Pharmacological doses of AA induce cell death by production of H_2_O_2_, but not by ferroptosis. **l** IncuCyte analysis showing that IKE + DHAA mediated death induction does not involve necroptosis, apoptosis or H_2_O_2_ production. In panels **k** and **l** the compounds were used at the following concentrations: Fer 5 μM; Trolox 100 μM; Catalase 200 U/mL; Nec-1 30 μM, Nec-1s 10 μM and Z-VAD-FMK 30 μM. Data are presented as mean ± SEM, from at least three independent biological replicates. ^***^P < 0.001; ^**^P < 0.01, ^*^P < 0.05, and n.s., not significant (*P* > 0.05).

Certain cell lines of tumor origin, such as prostate cancer cells (PCa) [19] and glioblastoma multiforme (GBM) cells [20], present as resistant to system x_c_^-^ inhibitors such as erastin and IKE. We found that GBM and PCa cells have high resistance to IKE-induced ferroptosis (Fig. 1a–d, Supplementary Fig. 1a). Strikingly, DHAA strongly sensitized a panel of GBM and PCa cells to ferroptosis mediated by the inhibition of system x_c_^-^ with IKE (Fig. 1b–d, Supplementary Fig. 1a). We corroborated that IKE + DHAA induced ferroptotic death in GBM and PCa through the pharmacological inhibition of lipid ROS accumulation with Fer and the recovery of cysteine uptake with 2-mercaptoethanol (β-ME). Finally, iron dependence was corroborated by chelating iron with deferoxamine (DFO) (Fig. 1e, f). However, we also identified two cell models in which treatment with Fer or Trolox does not rescue cells from death and dependence on cysteine is maintained, given that the use of NAC or βME effectively prevents cell death (Supplementary Fig 1b, c), similar to observations in other studies [21]. On another hand, where IKE + DHAA is a potent inducer of ferroptosis, it is tempting to speculate that IKE + DHAA is rapidly depleting GSH, favoring strong GPX4 inactivation. Corroborating this mechanism, when we measured the levels of GSH, we found that IKE + DHAA as a potent GSH depletory (Fig. 1g). However, when we pre-treated the cells with BSO for 24 h to deplete GSH, and then co-treated with DHAA, we did not observe induction of cell death (Fig. 1h). Thus, these data strongly suggest that in addition to a decrease in GSH levels, intracellular depletion of cysteine is necessary for DHAA-mediated induction of ferroptosis. This is supported by the fact that the absence of cystine in the culture medium and co-treatment with DHAA efficiently reproduces the effects of IKE + DHAA (Fig. 1i). Subsequently, we elucidated whether there was any crossover between the death induced by pharmacological doses of AA and ferroptosis induced by IKE + DHAA. However, only high doses of AA, but not DHAA, induced tumor death (Fig. 1j, Supplementary Fig 1d, e). Consistently, only catalase treatment prevented AA-mediated death, and Fer and Trolox had no protective effects (Fig. 1k, Supplementary Fig 1f). Furthermore, we and others have shown that AA favors the activation of necroptosis or apoptosis under some experimental conditions [4, 12, 22]. However, cell death induced by IKE + DHAA was not prevented by the caspase inhibitor Z-VAD-FMK, by the necroptosis inhibitor Nec-1s or by treatment with catalase, but it was effectively inhibited by Fer, Trolox, or high dose Nec-1 (Fig. 1l, Supplementary Fig. 1 g–k), which demonstrates the specificity for ferroptosis. It is important to note that DHAA was only synergistic in co-treatment with IKE to induce sensitization to ferroptosis, as our data show that DHAA has no potentiating effects on ferroptosis induction mediated by RSL3 or RSL3 + iFSP1 (Supplementary Fig 2). We believe that this effect is triggered because in the absence of IKE, DHAA can be efficiently reduced to AA due to the constant supplementation of cysteine, which would maintain high levels of GSH. In this context, intracellularly, AA is highly efficient in regenerating α-tocopherol [23], a lipophilic antioxidant capable of inhibiting ferroptosis [24]. Thus, we speculate that when the system x_c_^-^ is active, DHAA is intracellularly reduced to AA, which acts as an antioxidant and regenerates α-tocopherol, thereby inhibiting ferroptosis induced by RSL-3 or RSL-3 + iFSP1. In turn, our previous studies and those of others have shown that the recycling of DHAA to AA or the intracellular accumulation of AA is a potent inhibitor of cell death [16, 25, 26] as summarized in the diagram shown in Supplementary Fig 2k. These data strongly suggest that there is a DHAA-dependent mechanism that induces ferroptosis in tumor cells resistant to IKE, and that there is no relationship between ferroptosis and cell death induced by pharmacological doses of AA.

### IKE + DHAA induces ferroptosis in 3D tumor spheroids of GBM and PCa cells

**Fig. 2:**
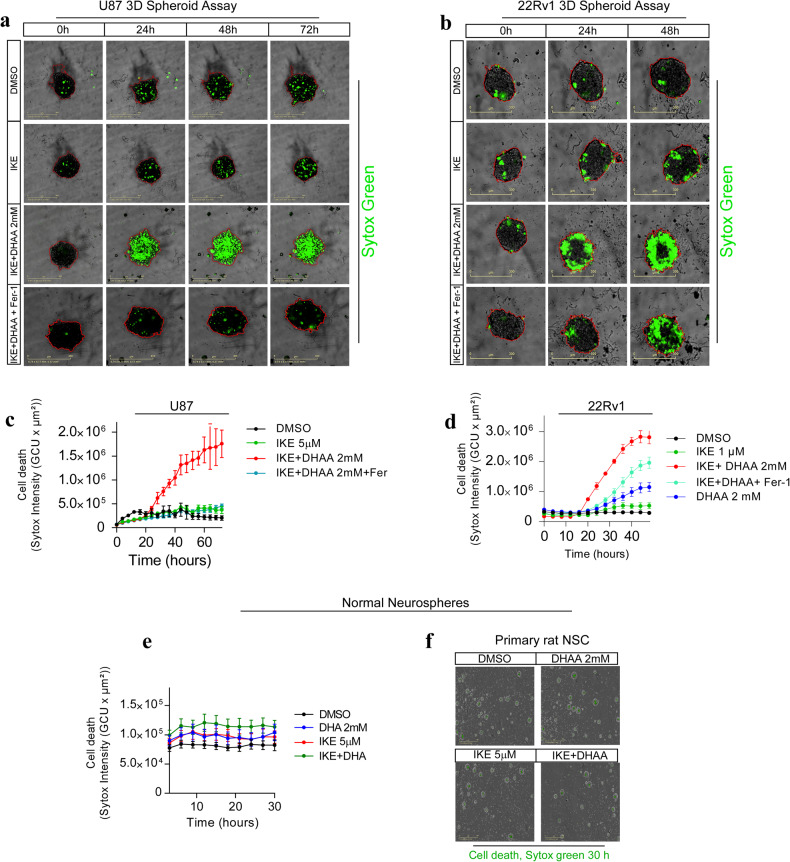
IKE + DHAA induces tumor death by ferroptosis in 3D models. **a**, **b** Death kinetics assays in 3D spheroids of U87 and 22Rv1 cells determined by IncuCyte and incorporation of Sytox green. **c**, **d** IKE + DHAA induces ferroptotic death in 3D models of GBM and PCa. **e**, **f** Treatment with IKE, DHAA (2 mM) or IKE + DHAA does not have toxic effects on primary neurospheres. Data are presented as mean ± SEM, from at least three independent biological replicates.

To emulate an in vivo context and to determine if our treatment is capable of eradicating 3D tumors, we cultured U87 and 22Rv1 cells in 3D spheroids. Consistently, IKE alone failed to induce death in 3D models of U87 and 22Rv1 cells (Fig. 2a–d). However, co-treatment with DHAA favored the induction of death in 3D spheroids of U87 and 22Rv1 cells, an effect that was rescued by treatment with Fer (Fig. 2a–d). To determine if the administered treatment presents toxic effects on normal cells, we cultured rat neural stem cells (NSCs) to simulate 3D culture conditions in normal neurospheres (Fig. 2e). When NSCs were incubated under the experimental conditions, there was no apparent effect (Fig. 2e, f), a finding that suggests that IKE + DHAA could be selective for the elimination of certain tumor cells.

### IKE + DHAA induces ferroptosis through lipid droplet peroxidation

**Fig. 3:**
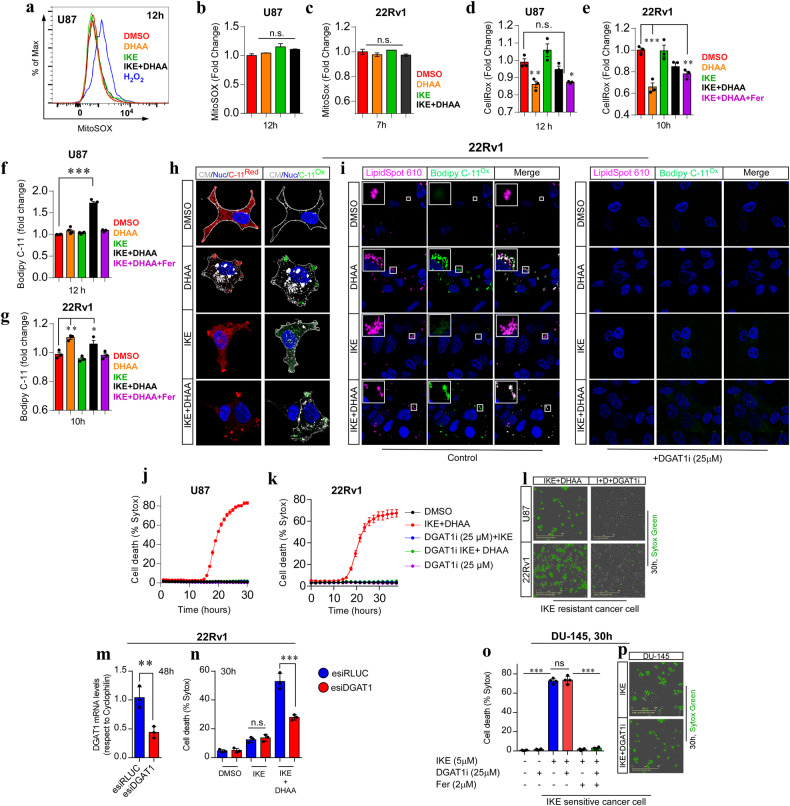
IKE + DHAA induces ferroptosis by peroxidation of lipid droplets. **a**, **b**, **c** Determination of mitochondrial ROS by FACS in U87 and 22Rv1 cells. **d**, **e** Determination of total ROS by FACS in U87 and 22Rv1 cells. **f**, **g** Determination of lipid ROS by FACS in U87 and 22Rv1. **h** Super-resolution live-cell microscopy using bodipy C-11 for lipid staining (green, oxidized lipids; red, reduced lipids), cellmask as a membrane marker (white) and Hoechst as a nuclear marker (blue), where it is observed that IKE + DHAA does not induce lipid peroxidation at the plasma membrane. **i** Super-resolution live-cell microscopy using bodipy C-11 for oxidized lipid staining (green), LipidSpot 610 for lipid droplet labeling (magenta) and Hoechst as nuclear marker (blue), where DHAA and IKE+ are observed DHAA induces lipid droplet peroxidation, which is completely inhibited by blocking DGAT1i. **j**, **k** IncuCyte analysis showing that inhibition of lipid droplet biosynthesis driven by DGAT1 blockade prevents IKE + DHAA-induced ferroptosis (*n* = 3 independent biological replicates). **l** Representative photomicrographs of **j** and **k**. **m** Validation of Dgat1 knockdown by qrt-PCR in 22Rv1 cells. **n** *Dgat1* knockdown inhibits IKE + DHAA-induced. **o**, **p** IncuCyte analysis showing that inhibition of lipid droplet biosynthesis driven by DGAT1 blockade does not prevent ferroptosis in IKE-sensitive cells. In all cases, DHAA was used at a concentration of 1 mM and IKE was used at 5 µM for U87 cells and 2 µM for 22Rv1 cells. Data are presented as mean ± SEM, from at least three independent biological replicates. ^***^P < 0.001; ^**^P < 0.01, ^*^P < 0.05; and n.s., not significant (*P* > 0.05).

Taking into account that this ferroptosis-triggering mechanism does not correspond to a previously described canonical pathway, we analyzed the production of mitochondrial, total, and lipid ROS in U87 and 22Rv1 cells. We did not detect mitochondrial ROS production in response to IKE + DHAA treatment (Fig. 3a–c). Despite this, we found that IKE + DHAA induced large mitochondrial fragmentation that was associated with a co-distribution with dynamin-related protein 1 (Drp-1) (Supplementary Fig. 3a) and decreased mitochondrial volume (Supplementary Fig. 3b). However, the inhibition of Drp-1 with Mdivi-1 did not rescue the cells from death (Supplementary Fig. 3c), suggesting that there was no direct link between Drp-1 and the induction of ferroptosis mediated by IKE + DHAA. Interestingly, we did not find a significant increase in the total ROS levels (Fig. 3d, e). However, in U87 cells, IKE + DHAA specifically induced lipid peroxidation, which was efficiently inhibited by Fer (Fig. 3f), a finding that would explain the activation of ferroptosis. Unexpectedly, treatment with DHAA alone was capable of inducing, similar to IKE + DHAA, a significant increase in lipid peroxidation in 22Rv1 cells (Fig. 3g). Thus, using super-resolution live-cell microscopy and the C11-BODIPY probe, we investigated the origin of IKE + DHAA-induced lipid peroxidation. Our live-cell super-resolution microscopy analyses revealed that IKE + DHAA did not induce lipid peroxidation in the plasma membrane (Fig. 3h, Supplementary Fig 3d). Strikingly, IKE + DHAA induced lipid peroxidation specifically in circular intracellular structures (Fig. 3h, Supplementary Fig 3d) which we speculated could be lipid droplets. Lipid droplets inhibit the induction of ferroptosis in some cancer cells [27]. Therefore, the induction of lipid droplet peroxidation could be an interesting and novel target for triggering ferroptosis. To corroborate this hypothesis, we used the lipid droplet stain LipidSpot 610 and the C11-BODIPY probe to determine if lipid droplet peroxidation was present. Confirming our hypothesis, live-cell images showed that IKE + DHAA specifically induces lipid droplet peroxidation (Fig. 3i, Supplementary Fig 3e). Reinforcing this finding, the depletion of lipid droplets driven by the inhibition of diacylglycerol-acyltransferase 1 (DGAT1) completely prevented lipid peroxidation induced by DHAA or by IKE + DHAA (Fig. 3i). Additionally, the blockade of lipid droplet synthesis mediated by the inhibition of DGAT1 completely prevented ferroptosis induced by IKE + DHAA in U87 and 22Rv1 cells (Fig. 3j, k, l). To confirm the specificity of this pathway, we silenced the expression of DGAT1 by using esiRNA (Fig. 3m). Consistently, DGAT1 silencing efficiently inhibited IKE + DHAA-induced ferroptosis (Fig. 3n). It is important to highlight that this ferroptosis induction mechanism through lipid droplet peroxidation was selective for cells that have resistance to IKE; when we use DU-145 cells that are sensitive to IKE as positive control, inhibition of DGAT1 fails to block ferroptosis (Fig. 3o, p). These results corroborate that lipid droplets are dispensable for induction of ferroptosis in IKE-sensitive models, similar to other reports [28]. But peroxidation of lipid droplets are key in cancer models that have resistance to IKE, to be sensitized to ferroptosis through co-treatment by IKE + DHAA.

### IKE + DHAA depletes GPX4 and induces GPX4 delocalization from lipid droplets in GBM and PCa cells

**Fig. 4:**
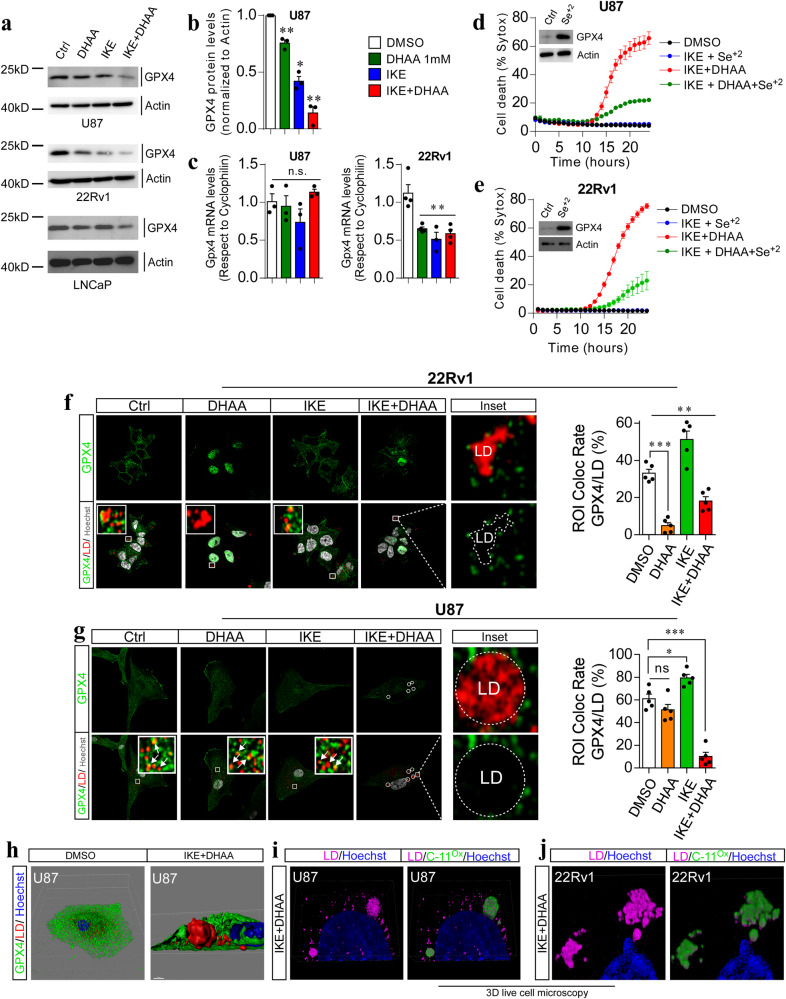
IKE + DHAA induces depletion and delocalization of GPX4 from lipid droplets. **a** Western blot analysis of GPX4 abundance. **b** Quantification of GPX4 abundance in U87 cells. **c** qRT-PCR of GPX4 mRNA levels in U87 and 22Rv1 cells at 7 and 5 h post-treatment, respectively. **d**, **e** IncuCyte analysis showing that GPX4 overexpression inhibits IKE + DHAA-induced ferroptosis. **f** Lightning super-resolution microscopy showing GPX4 delocalization in lipid droplets in 22Rv1. **g** Lightning super-resolution microscopy showing GPX4 delocalization and inaccessibility in lipid droplets in U87. **h** 3D reconstruction of the large lipid droplet and GPX4 in U87 cells. **i**, **j** 3D live cell microscopy showing that large lipid droplets are specifically peroxidized in U87 and 22Rv1 cells. Data are presented as mean ± SEM, from at least three independent biological replicates. ^***^P < 0.001, ^**^P < 0.01, ^*^P < 0.05, and n.s., not significant (*P* > 0.05).

To reveal a better understanding of the mechanism by which ferroptosis is induced by IKE + DHAA in GBM and PCa cells, we performed a detailed analysis of GPX4 levels in both cell models and determined that DHAA alone and IKE + DHAA induced a decrease in GPX4 levels in GBM and PCa cells (Fig. 4a, b, c). To corroborate the importance of the decrease in GPX4 levels in facilitating ferroptosis, we induced GPX4 accumulation by pre-treatment with selenium, which rescued GBM and PCa cells from death induced by IKE + DHAA (Fig. 4e). Strikingly, we found that IKE + DHAA specifically induced delocalization of GPX4 from lipid droplet (Fig. 4f, g). In a similar manner, in 22Rv1 and U87 cells we detected that IKE + DHAA induced the formation of abnormally large lipid droplets (Fig. 4f, g (inset), h), similar to the effect of cysteinase observed in pancreatic tumors [29], where we speculate that GPX4 cannot access lipid droplets to inhibit lipid peroxidation. In line with this notion, these large lipid droplets are the ones we find specifically peroxidized (Fig. 4i, j). In this scenario, these results show that in addition to the depletion of GPX4, delocalization/inaccessibility of this protein to lipid droplets must occur to favor its peroxidation and trigger ferroptosis in cancer cells resistant to IKE.

### IKE + DHAA eradicates glioblastoma in vivo

**Fig. 5:**
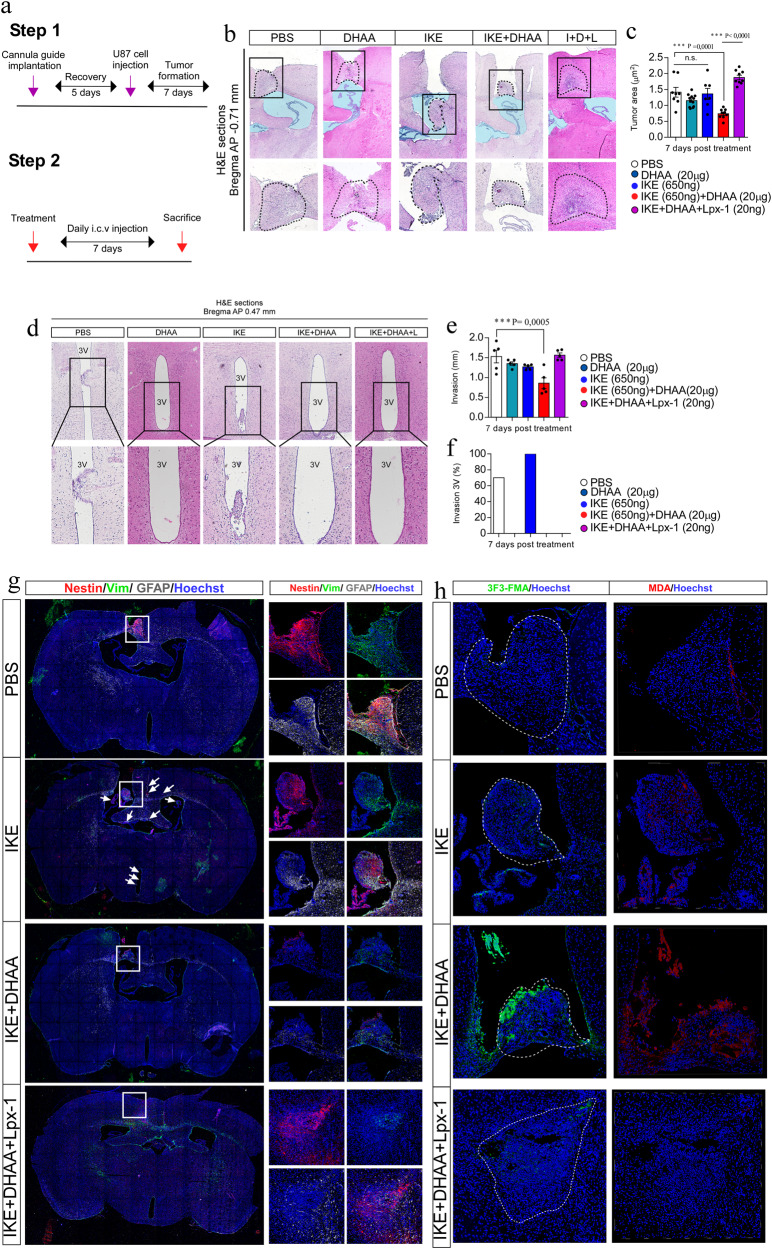
Intratumorally injection of IKE + DHAA induces GBM eradication. **a** Scheme of the tumor induction and treatment protocol. **b** Histological analysis by H&E of the effect of IKE and IKE + DHAA at 7 days post treatment. **c** Tumor area quantification. *n* = 7–12 mice per condition. Each point represents a brain slice from a different analyzed animal. **d** H&E analysis of third ventricle invasion. **e** Quantification of tumor invasion in the anteroposterior axis. *n* = 5 mice per condition. **f** Quantification of GBM cell invasion of the third ventricle. *n* = 5 mice per condition **g** Confocal microscopy analysis of GBM cells in vivo. **h** Super-resolution analysis for the identification of ferroptotic cells (3F3-FMA stain, green) and lipid peroxidation (MDA stain, red). Data are presented as mean ± SEM. ^***^P < 0.01, ^**^P < 0,01, ^*^P < 0.05; and n.s., not significant (*P* > 0.05).

To determine whether IKE + DHAA has efficacy in vivo, we generated xenographs of U87 cells. Thus, using convection-enhanced delivery (CED) to bypass the blood brain barrier (BBB) [30], we intracerebroventricularly (icv) injected PBS, DHAA, IKE, IKE + DHAA or IKE + DHAA+Liproxstatin (Lpx) (Fig. 5a). Then, from day 7, we injected saline solution (PBS), DHAA (20 μg), IKE (650 ng), IKE + DHAA or IKE + DHAA+Lpx (20 ng) icv directly into the tumor injection site through a previously implanted cannula guide daily for 1 week (Fig. 5a). Our data showed that the icv injection of IKE or DHAA alone failed to reduce tumor size and invasion (Fig. 5b, e). Moreover, treatment with IKE increased tumor invasion into other areas, such as the third ventricle (3V) (Fig. 5d, f). However, daily treatment with IKE + DHAA decreased tumor size and prevented invasion and migration to 3V (Fig. 5b–f), an effect that was efficiently inhibited by the administration of Lpx, corroborating that IKE + DHAA can induce ferroptosis in vivo (Fig. 5b, e). On the other hand, when performing immunohistochemistry using Nestin as a marker to identify GBM cells [31, 32], tumor cells (Nestin + ) presented strong reactive gliosis given the co-distribution of Vimentin (green) and GFAP (white) in the control, IKE and IKE + DHAA+Lpx conditions in the tumor area (Fig. 5g,). Unexpectedly, treatment with IKE induced the migration of Nestin+ cells to various parenchymal areas and to the third ventricle (Fig. 5g, arrow), a finding that suggests that in response to IKE, GBM cells “escape” the tumor area by invading other regions, eventually increasing the aggressiveness of the GBM, similar to what occurs in patients treated with sulfasalazine [33]. Fortunately, IKE + DHAA through CED resulted in a dramatic decrease in Nestin^+^ cells, suggesting GBM eradication after 7 days of treatment (Fig. 5g), effect that was prevented by the administration of Lpx (Fig. 5g). Furthermore, IKE + DHAA treatment decreased Vimentin and GFAP staining, suggesting a decrease in reactive gliosis (Fig. 5g, green and white stain). Furthermore, using the 3F3-FMA antibody, which recognizes ferroptotic cells [34], we observed that IKE + DHAA induces a strong TfR signal in the tumor area (Fig. 5h), corroborating that IKE + DHAA favors the induction of ferroptosis in vivo, because Lpx treatment prevents 3F3-FMA immunoreactivity (Fig. 5h). Finally, we confirm that IKE + DHAA induces a strong peroxidation of lipids in vivo specifically in the tumor area using Anti-Malondialdehyde (MDA), an effect that was efficiently inhibited by Lpx administration (Fig. 5h). Thus, our data strongly suggest that the local injection of IKE + DHAA induce ferroptosis in vivo in the tumor area and could have highly beneficial effects for the eradication of highly aggressive tumors.

## Discussion

Our study reveals unexplored crosstalk between the oxidized form of vitamin C, DHAA, and the inhibition of system x_c_^-^ that can be exploited to favor the induction of ferroptosis in cancer cells that are difficult to treat and that present resistance to IKE-induced ferroptosis. It is important to highlight, that our data confirm previous publications where it has been shown that there is no relationship between ferroptosis and cell death induced by pharmacological doses of AA, which is restricted to extracellular H_2_O_2_ overproduction [12, 13, 35]. In line with this notion, our data show that there is no relationship between AA-induced death and ferroptosis induction by treatment with IKE + DHAA. Thus, special precaution should be taken when using high doses of AA to erroneously conclude activation of ferroptosis [36, 37]. This misinterpretation of data is possible when specific controls are not used to selectively inhibit ferroptosis as ferrostatin-1 or liproxtatin-1 [36, 37]. These controls are key, since there are other types of death that are iron-dependent or involve lipid peroxidation that are not related to ferroptosis [8, 38, 39].

Regarding the mechanism by which IKE + DHAA sensitizes tumor cells to ferroptosis, we found that it involves depletion of GSH and induction of lipid droplet (LD) peroxidation without inducing lipid peroxidation in the plasma membrane. Contrary to other studies in which the depletion of LDs due to the inhibition of DGAT1 confers sensitization to apoptosis [40] or ferroptosis in cancer models [27], in this study, the inhibition of DGAT1 blocks ferroptosis induced by IKE + DHAA selectively in IKE-resistant cancer models. Importantly, our data establish that LD peroxidation could be due to a decrease in GPX4 levels, which must be accompanied by an “inaccessibility or loss of localization” of GPX4 in the LD. However, it is unknown how or why IKE + DHAA generates these alterations in GPX4 localization. We can speculate that lipid droplets can act as a reservoir of PUFAs and PUFA-PLs in some cancer cells, rendering them resistant to ferroptosis. In such a scenario, DHAA may be able to gain access to lipid droplets and induce oxidation and inactivation of GPX4, which is highly susceptible to oxidation and subsequent degradation.

Finally, our data show that IKE + DHAA is highly effective in vivo for the eradication of GBM through the induction of tumor-cell specific ferroptosis via local therapy with stereotaxic injections through CED, an approach that could be a new, highly efficient battlefront against GBM [30]. In this scenario, it is important to highlight that drug administration via CED has been successfully tested in humans in phase I clinical trials to treat GBM and overcome the BBB [41–43]. However, given the surgical difficulty of this procedure in patients, its widespread application is limited [30]. In this context, administration of DHAA in concentrations greater than 1 mM in animals or humans has been shown to be safe, well tolerated and without any adverse effect [44, 45]. Furthermore, Slc7a11-deficient animals develop and reproduce in a completely normal manner [46]. This suggests that treatment with DHAA and system x_c_^-^ inhibition mediated by IKE would not have major adverse effects. However, caution should be used when administering IKE for the treatment of GBM because our in vivo experiments showed that the administration of this compound favors GBM migration, similar to the findings of a clinical trial of sulfasalazine that had to be suspended [33].

Together, our data strongly suggest that IKE + DHAA is a potent inducer of ferroptosis in cancer cells that are resistant to IKE, with potential in vivo applications.

### Reporting summary

Further information on research design is available in the Nature Research Reporting Summary linked to this article.

### Supplementary information

original data files

Ferrada et at Supplemental Material

Reporting Summary

## Data Availability

All data necessary for reproducibility are contained in this manuscript. Any other additional information is available upon request to the corresponding author.
