# Supplementary figures and images for: Dehydroascorbic acid sensitizes cancer cells to system xc- inhibition-induced ferroptosis by promoting lipid droplet peroxidation
Source: Cell Death Dis. 2023 Sep 27;14(9):637. doi: 10.1038/s41419-023-06153-9 (PMC10522586; doi:10.1038/s41419-023-06153-9)

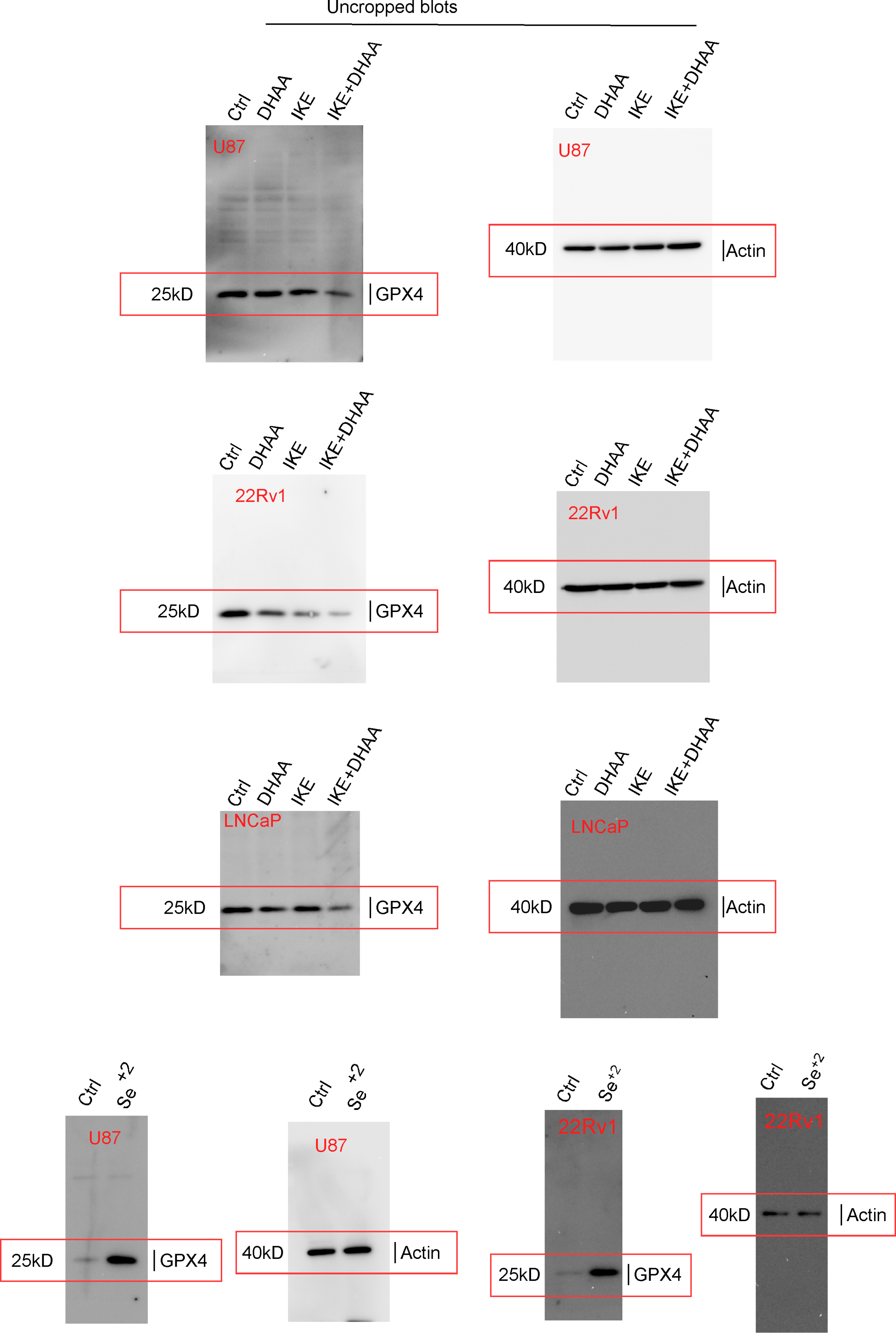

Supplement: Supplementary file 1 — original data files [file 41419_2023_6153_MOESM1_ESM.docx]
