# Supplementary material for: Dehydroascorbic acid sensitizes cancer cells to system xc- inhibition-induced ferroptosis by promoting lipid droplet peroxidation
Source: Cell Death Dis. 2023 Sep 27;14(9):637. doi: 10.1038/s41419-023-06153-9 (PMC10522586; doi:10.1038/s41419-023-06153-9)

Supplementary Materials for

Dehydroascorbic acid sensitizes cancer cells to system xc- inhibition-induced ferroptosis by promoting lipid droplet peroxidation.

Luciano Ferrada[1*], María José Barahona[1,2], Matías Vera[1], Brent R. Stockwell[3,4] and Francisco Nualart[1,2]

*Corresponding author. Email: luferrada@udec.cl

This PDF file includes:

Key resources table

Figures. S1 to S3

Uncropped Blots

Table 1. Key resources table

REAGENT or RESOURCE	SOURCE	IDENTIFIER
**Antibodies**		
β-Actin (C4)	Santa Cruz Biotech	sc-47778 HRP
GPX4 (E-12)	Santa Cruz Biotech	sc-166570
Drp-1	Santa Cruz Biotech	sc-271583
GFAP	DAKO	#Z0334
Vimentin	Merck	#AB5733
MDA (1F83)	Adipogen	JAI-MMD-030N
3F3-FMA	Stockwell lab	(Feng et al., 2020)
**Chemicals and compounds**		
Sytox green	Invitrogen	# S7020
Cellmask	Invitrogen	# C10046
Lipid Spot 610	Biotium	#70069
Erastin	Merck	#E7781
RSL-3	Merck	#SML2234
Ferrostatin-1	Merck	#SML0583
L-Ascorbic Acid	Merck	#A4403
Dehydroascorbic Acid (DHAA)	Merck	#D8132
Catalase	Merck	#C1345
Necrostatin-1	Merck	#N9037
Necrostatin-1s	BioVision	#2263
2-mercaptoethanol	GIBCO	#21985023
DFO	Cayman	# 14595
iFSP1	Cayman	#29483
BSO	Cayman	#14484
A 922500 (DGAT1i)	MedChemExpress	# HY-10038
Z-VAD-FMK	MedChemExpress	#HY-16658B
Trolox	MedChemExpress	# HY-101445
Mdivi-1	Abcam	#ab144589
Liproxstatin	MedChem	#HY-12726
IKE (in vivo)	MedChem	#HY-114481
IKE (in vitro)	Stockwell lab	n/a
**Critical commercial assays**		
Brilliant II SYBR® Green QPCR Master Mix	Agilent	#600828
High Capacity cDNA Reverse Transcription kit	Applied Biosystems	#4374967
Image-iT™ Lipid Peroxidation Kit	Invitrogen	#C10445
CellROX™ Deep Red Reagent	Invitrogen	#C10422
MitoSox	Invitrogen	#M36008
Trizol	Invitrogen	#15596018
Mitotracker Red CMXRos	Invitrogen	#M7512
CellMask deepRed	Invitrogen	#C10046
RNAiMAX	Invitrogen	#13778075
LipidSpot 610	Biotium	#70069
GSH Assay Kit	Abcam	#ab239727
**Experimental models: Cell lines**		
U87	ATCC	#HTB-14

HSVT-C3	(Jara et al., 2021)	n/a
Brain Tumor Initiating Cells Patient 13 (BTIC-13)	(Ramirez et al., 2022)	n/a
LNCaP	ATCC	#CRL-1740
C4-2B	ATCC	#CRL-3315
22Rv1	ATCC	#CRL-2505
DU-145	ATCC	#HTB-81
ZR-75-30	ATCC	# CRL-1504
HeLa	ATCC	# CRM-CCL-2
Human Dermal Fibroblasts	ThermoFisher	# C0135C
**Experimental models: Organisms/strains**		
C57BL/6j	The Jackson Laboratory	IMSR_JAX:000664
**Oligonucleotides**		
esiRNA RLUC (Ctrl)	Merck	EHURLUC
esiRNA Human DGAT1	Merck	EHU147001
**qRT-PCR Primers**		
GPX4 Fw	CAGCTTGCGACCGGAG	This paper
GPX4 Rv	CCGAACTGGTTACACGGGA	This paper
DGAT1 Fw	CGAGGGTGTCAATAAAGTGCTG	This paper
DGAT1 Rv	TCTGGCACTCGCCCTTG	This paper
Cyclophilin Fw	ATAATGGCACTGGTGGCAAGTC	This paper
Cyclophilin Rv	ATTCCTGGACCCAAAACGCTCC	This paper
**Software and algorithms**		
Incucyte® Cell-by-Cell Analysis Software	Sartorius	# 9600-0031
Incucyte® Spheroid Analysis Software	Sartorius	# 9600-0019
LASX with Lightning module	Leica	n/a
Imaris	Bitplane	n/a
Opentrons protocol designer	https://designer.opentrons.com	n/a

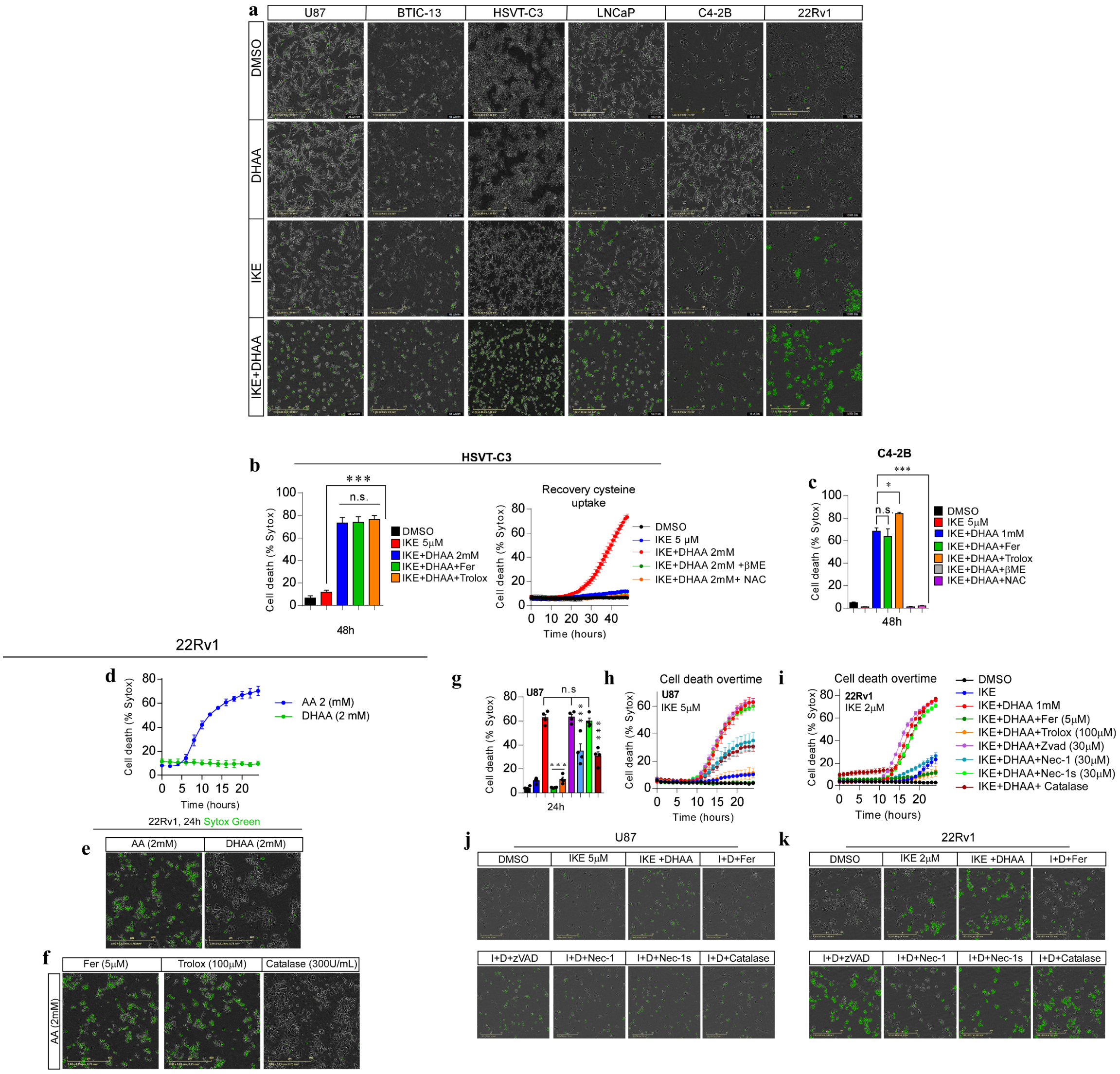

Fig. S1. IKE+DHAA induces ferroptosis in cancer cells.

a. Representative microphotographs of the effect of IKE+DHAA on GBM and PCa cells, the green staining corresponds to the incorporation of Sytox green. **b, c.** IKE+DHAA induces cysteine uptake-dependent death, which is not rescued by lipid peroxidation inhibitors. **d.** Effect of AA or DHAA on the induction of cell death in 22Rv1 cells. **e, f.** Representative photomicrographs of AA effect and ferroptosis and no ferroptosis inhibitors. **g, h, i.** Inhibitory effect of different death suppressors on ferroptosis induced by IKE+DHAA. **j, k.** Representative microphotographs of the effect of the

different death suppressors. Compounds were used in the following concentrations: Fer 5 μM; Trolox 100 μM; Catalase 200U/mL; Nec-1 30μM, Nec-1s 10 μM, Zvad 30 μM, βME 50μM, NAC 1mM. Data are presented as mean ± SEM, from at least three independent biological replicates. ***P < 0,001; **P<0.01 *P < 0.05; and n.s., not significant (P > 0.05) (one-way ANOVA).

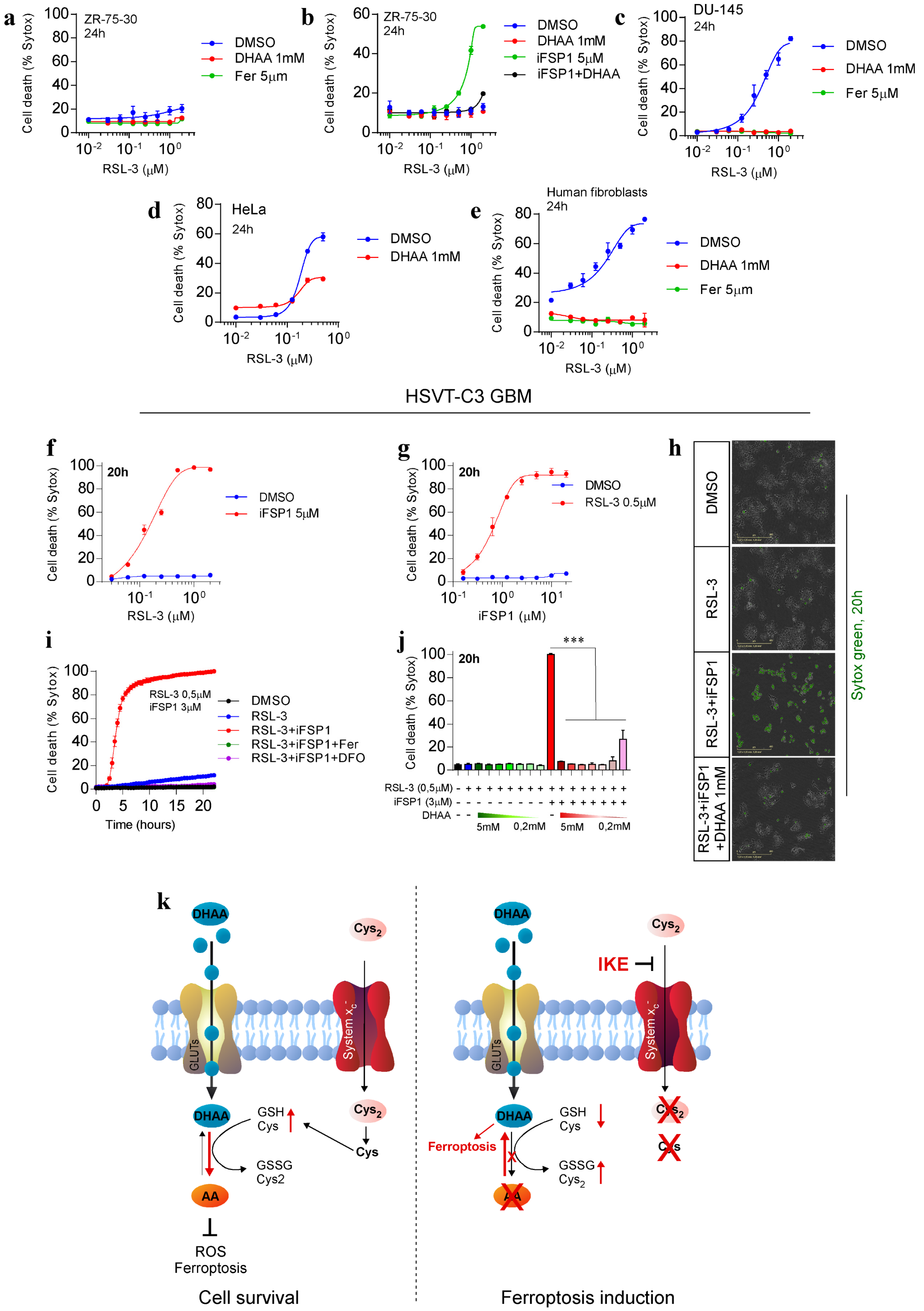

a
ZR-75-30
24h
DMSO
DHAA 1mM
Fer 5µm
Cell death (% Sytox)
RSL-3 (µM)
b
ZR-75-30
24h
DMSO
DHAA 1mM
iFSP1 5µM
iFSP1+DHAA
c
DU-145
24h
DMSO
DHAA 1mM
Fer 5µM
d
HeLa
24h
DMSO
DHAA 1mM
e
Human fibroblasts
24h
DMSO
DHAA 1mM
Fer 5µm
HSVT-C3 GBM
f
20h
DMSO
iFSP1 5µM
g
20h
DMSO
RSL-3 0.5µM
iFSP1 (µM)
h
DMSO
RSL-3
RSL-3+iFSP1
RSL-3+iFSP1 +DHAA 1mM
Sytox green, 20h
i
RSL-3 0,5µM
iFSP1 3µM
DMSO
RSL-3
RSL-3+iFSP1
RSL-3+iFSP1+Fer
RSL-3+iFSP1+DFO
Time (hours)
j
20h
***
RSL-3 (0,5µM)
iFSP1 (3µM)
DHAA
5mM
0,2mM
5mM
0,2mM
k
DHAA
Cys2
GLUTs
System xc-
GSH
Cys
GSSG
Cys2
AA
ROS
Ferroptosis
Cell survival
IKE
Ferroptosis
Ferroptosis induction

Figure S2. DHAA is a potent inhibitor of ferroptosis induced by RSL-3 or RSL-3+ iFSP1.

a-e. Dose-response curve showing that DHAA inhibits the induction of ferroptosis mediated by RSL-3 in different cell models. **f.** IncuCyte analysis showing that iFSP1 co-treated with RSL-3 sensitizes GBM cells to induction of cell death. **g.** IncuCyte analysis showing the dose-response curve of the effect of iFSP1 on the induction of death mediated by co-treatment with RSL-3 in GBM cells. **h.** Representative photomicrographs of the induction of ferroptosis by RSL-3+iFSP1 and the inhibitory effect of DHAA on cell death in GBM. **i.** RSL-3+iFSP1 induces ferroptotic death in GBM cells. **j.** DHAA inhibits RSL-3+iFSP1-induced ferroptosis. **k.** Simplified diagram of the effect of DHAA and IKE+DHAA on cancer cells: Under normal conditions, cancer cells can efficiently recycle DHAA to AA intracellularly due to the constant supply of cysteine provided by the activity of the system x_c^-, which inhibits oxidative stress and ferroptosis (left panel). On the other hand, when cancer cells are treated with IKE+DHAA, DHAA cannot be reduced, resulting in an abrupt depletion of GSH and reducing power, favoring the induction of ferroptosis (right panel). Data are presented as mean ± SEM, from at least three independent biological replicates.

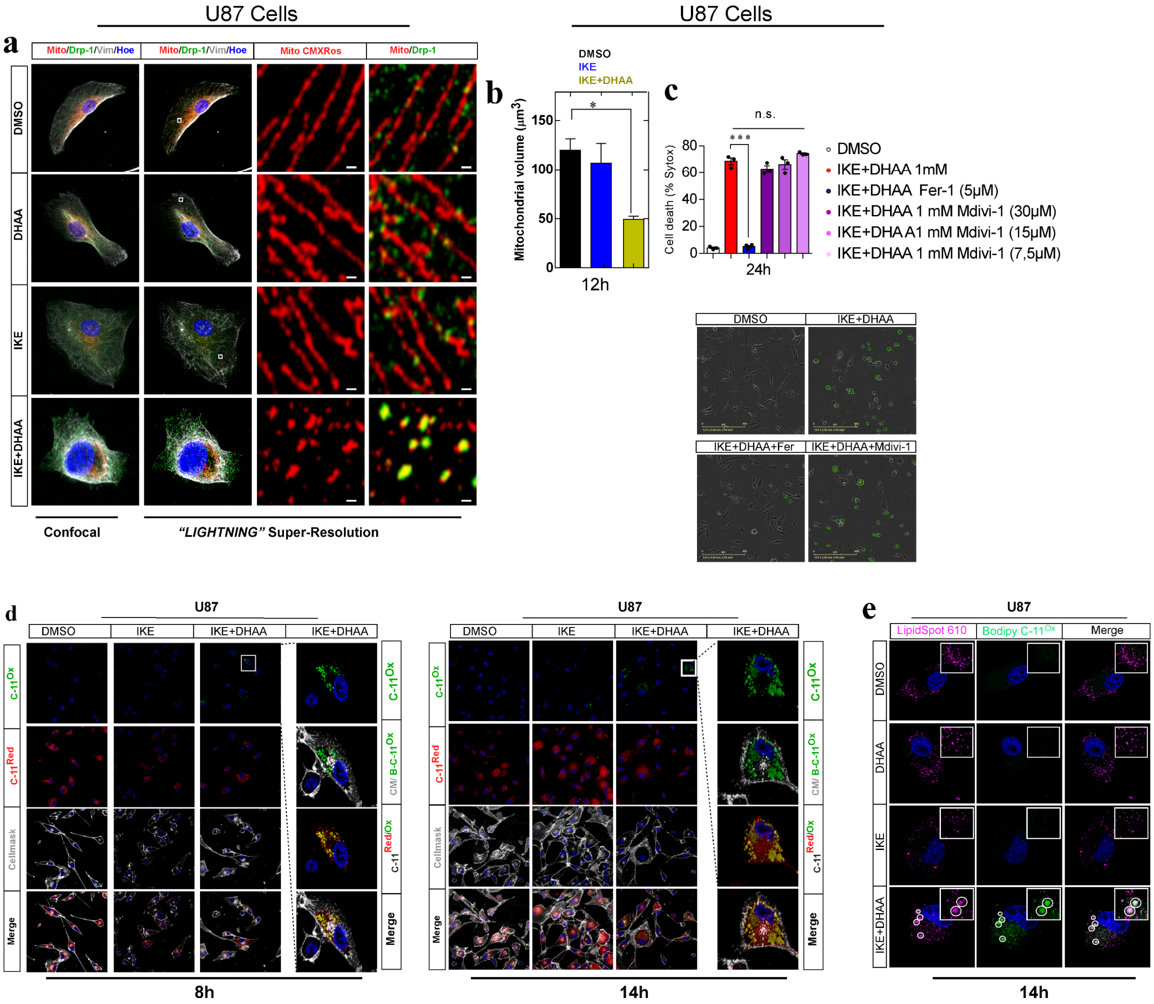

Figure S3. IKE+DHAA induces mitochondrial fragmentation and peroxidation of lipid droplets in U87 cells.

a. Super resolution microscopy analysis of mitochondrial morphology. **b.** Quantification carried out in Imaris of the mitochondrial volume. **c.** Effect of the inhibition of Drp-1 with Mdivi-1 on the ferroptosis induced by IKE+DHAA determined by IncuCyte. **d.** Super resolution live cell microscopy of lipid peroxidation in U87 cells at 8 and 14h post treatment. **e.** Super resolution live cell microscopy of lipid droplet peroxidation in U87 cell, in magenta the lipid droplets are observed, in green the oxidized lipids. The circles highlight clusters of peroxidized lipid droplets, which are closely related to the inaccessibility of GPX4 to these structures. (Data are presented as mean ± SEM, from at least

three independent biological replicates. ***P < 0.001; *P < 0.05; and n.s., not significant (P > 0.05) (one-way ANOVA).

Uncropped blots

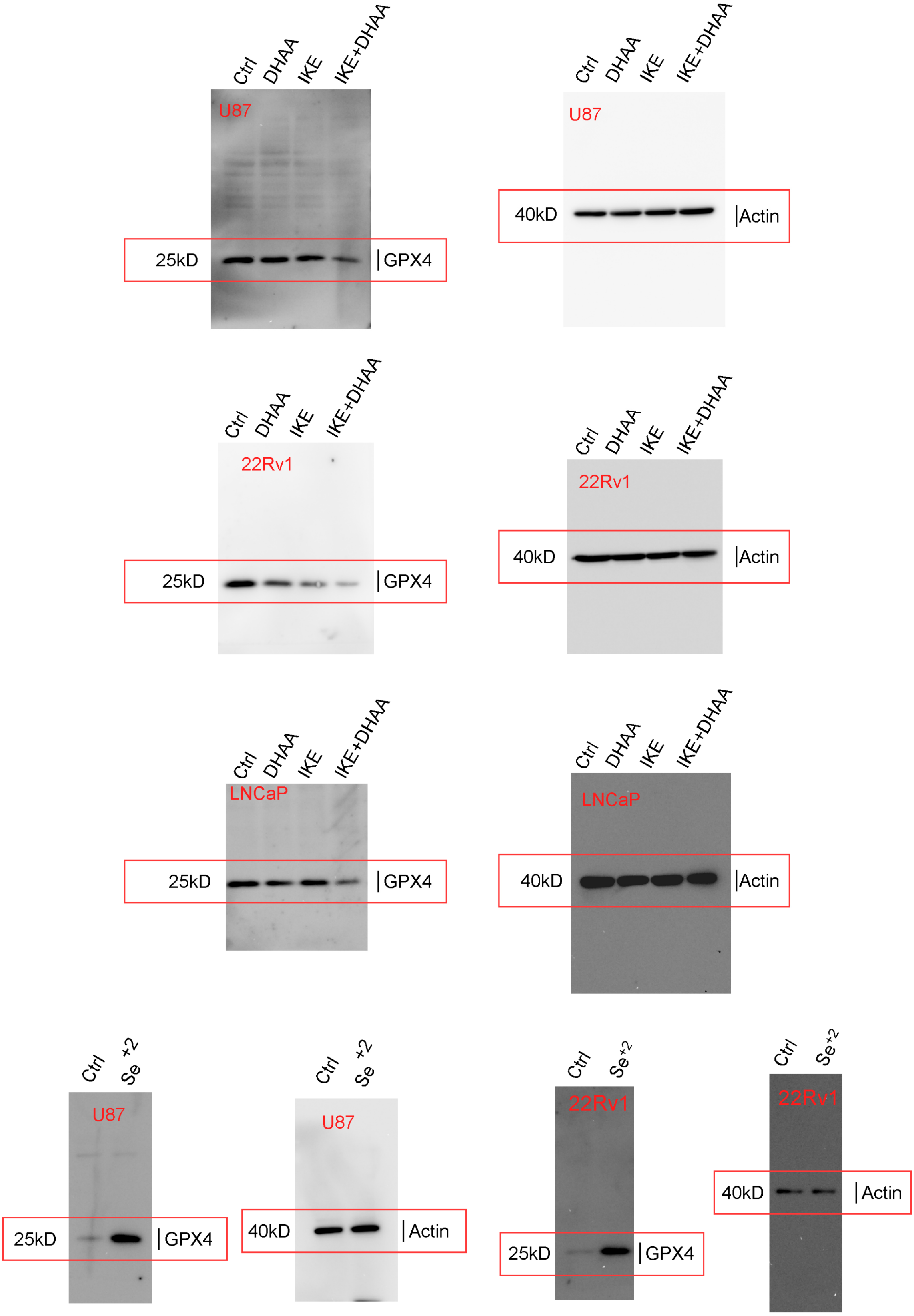

Supplement: Supplementary file 2 — Ferrada et at Supplemental Material [file 41419_2023_6153_MOESM2_ESM.pdf]
